# Aptamer Laden Liquid Crystals Biosensing Platform for the Detection of HIV-1 Glycoprotein-120

**DOI:** 10.3390/molecules26102893

**Published:** 2021-05-13

**Authors:** Amna Didar Abbasi, Zakir Hussain, Kun-Lin Yang

**Affiliations:** 1 Department of Materials Engineering, School of Chemical and Materials Engineering (SCME), National University of Sciences and Technology (NUST), Islamabad 44000, Pakistan; amna.phd@scme.nust.edu.pk; 2 Department of Chemical and Biomolecular Engineering, National University of Singapore, Engineering Drive 4, Singapore 117576, Singapore; cheyk@nus.edu.sg

**Keywords:** liquid crystals, RNA aptamer, B40t77, gp-120, DMOAP, biosensor

## Abstract

We report a label-free and simple approach for the detection of glycoprotein-120 (gp-120) using an aptamer-based liquid crystals (LCs) biosensing platform. The LCs are supported on the surface of a modified glass slide with a suitable amount of B40t77 aptamer, allowing the LCs to be homeotropically aligned. A pronounced topological change was observed on the surface due to a specific interaction between B40t77 and gp-120, which led to the disruption of the homeotropic alignment of LCs. This results in a dark-to-bright transition observed under a polarized optical microscope. With the developed biosensing platform, it was possible to not only identify gp-120, but obtained results were analyzed quantitatively through image analysis. The detection limit of the proposed biosensing platform was investigated to be 0.2 µg/mL of gp-120. Regarding selectivity of the developed platform, no response could be detected when gp-120 was replaced by other proteins, such as bovine serum albumin (BSA), hepatitis A virus capsid protein 1 (Hep A VP1) and immunoglobulin G protein (IgG). Due to attributes such as label-free, high specificity and no need for instrumental read-out, the presented biosensing platform provides the potential to develop a working device for the quick detection of HIV-1 gp-120.

## 1. Introduction

To date, success in developing an accurate, reliable and cost-effective human immunodeficiency virus type 1 (HIV-1) detection system has been obscure. Glycoprotein-120 (gp-120) is the envelope glycoprotein of HIV-1, which plays an important role in HIV-1 infection [1,2]. Certain features make gp-120 an important diagnostic marker in HIV-1 diagnostics, such as initial docking between the virus and human T cell membrane, shedding from the virus surface after docking and having well-conserved sequences between different HIV-1 mutants [3,4,5,6]. The present method to detect gp-120 is an enzyme-linked immunosorbent assay (ELISA), which is time consuming, requires additional labelling and many reaction steps to detect antibodies [5]. Therefore, a new cost-effective biosensing platform for simple and fast detection of gp-120 is much needed.

Recently, aptamer-based liquid crystal biosensors have gained substantial interests as new, label-free optical biosensing techniques owing to high sensitivity, affordability, fast detection, wide response range and ease of operation [7,8]. Liquid crystals (LCs) are a state of matter with fluidity, sensitive orientational response, elastic strain and birefringence. These properties make LCs an ideal candidate for biosensor fabrication [9]. Owing to orientational behavior and the low anchoring energy of LCs, they have been extensively exploited to amplify and transduce the interaction between aptamer and its target into optical signals through a cross-polarizer, which can be observed under a polarized optical microscope [10,11,12,13,14,15,16]. LC biosensing approaches are label-free and can detect various analytes with a simple readout, thus avoiding the problems related to present diagnostic techniques and offering several advantages over other conventional sensing techniques [17,18,19].

Aptamers are synthetic single-stranded oligonucleotides (DNA or RNA) with promising properties over antibodies, including high specificity, binding affinity, thermal and chemical stability, reusability, low immunogenicity, affordability and can be easily modified [20,21,22,23]. These remarkable properties make aptamers ideal candidates to use as recognition elements while designing a bio-sensing platform. Since aptamers can be designed for a wide variety of targets, they allow detection of both early and late infection markers [24]. Aptamers have been extensively used in biosensing technology for the detection of various targets, such as antibiotics, heavy metals, toxins, proteins, drugs and a wide variety of other contaminants [7,12,16,25,26,27,28,29]. Dey et al. reported B40t77 RNA aptamer for gp-120 in 2005 [30]. This aptamer can specifically interact with gp-120, resulting in the blockage of interaction between gp-120 and CCR5 receptors, which leads to neutralized infectivity of HIV-1 virus [31]. Over the past decade, researchers have been using aptamers frequently in diagnostics as recognition elements for biosensors fabrication, which were previously designed for therapeutics [32]. This approach has the possibility of paving the way for a new paradigm in HIV diagnostics.

Herein, for the first time, we report a label-free aptamer-based LC biosensing platform for gp-120 detection. In this work, a B40t77 aptamer was immobilized on a *N*,*N*-dimethyl-*n*-octadecyl-3aminopropyltrimethoxy-silyl chloride (DMOAP)-coated glass slide as the recognition element. The interaction between aptamer and target gp-120 protein disturbs the homeotropic alignment of LC molecules leading to a transition in the LCs optical appearance. This orientational transition of LCs can easily be observed in real time under a polarized optical microscope, in terms of switching between dark to bright optical images. The developed biosensing platform demonstrates huge potential to be explored further for the fabrication of a point-of-care device for the quick detection of HIV-1, without the need for a sophisticated lab and expert manpower.

## 2. Experimental Section

### 2.1. Materials

*N*,*N*-dimethyl-*n*-octadecyl-3aminopropyltrimethoxy-silyl chloride (DMOAP), bovine serum albumin (BSA), human immunoglobulin protein (IgG), hepatitis A virus capsid protein VP1 (Hep A VP1), RNase free water and magnesium chloride were purchased from Sigma Aldrich (Singapore, Singapore). Microscopic glass slides were purchased from Marienfield (Lauda-Königshofen, Germany). Liquid crystal 4-cyano-4-pentylbiphenyl (5CB) was obtained from Merck (Tokyo, Japan). Tris EDTA (1 × TE, pH-8) and phosphate-buffered saline (PBS) buffer (10×, pH-7.4) were procured from 1st BASE (Singapore, Singapore). Decon-90 was obtained from VWR (Singapore, Singapore). Mylar transparency film was obtained from Infinite Graphics (Singapore, Singapore). Protein gp-120 was purchased from Abcam (Cambridge, UK). 2-Fluoro pyrimidine substituted 77 nucleotides long B40t77 RNA aptamer was custom synthesized by Gene Link (Elmsford, NY, USA). All the stock and working solutions of gp-120, IgG, HepA VP1 and BSA were prepared by dissolving them in 0.01 M phosphate-buffered saline (PBS, pH 7.4). Aptamer stock solution was prepared by dissolving B40t77 in RNase-free water at room temperature and diluted by using TE buffer with 100 mM of magnesium chloride. Prior to use, the aptamer solution was heated to 95 °C for 3 min, cooled at room temperature for 5 min, and then cooled on ice to ensure correct spatial folding of the aptamer. All buffer solutions were prepared in 0.2 µm filtered milli Q water.

### 2.2. Cleaning and Chemical Modification of Glass Slides

Glass slides were first rinsed with deionized water two times and then immersed in a 5% (*v*/*v*) Decon-90 solution overnight. The slides were then cleaned ultrasonically in deionized water for 15 min and rinsed with copious amounts of deionized water. To prepare DMOAP-coated glass slides, the cleaned slides were then immersed in a 0.1% DMOAP solution (*v*/*v*) for 5 min. Subsequently, the slides were rinsed with deionized water five times to remove unreacted DMOAP from the surface and then dried under a compressed nitrogen gas stream. Finally, these slides were placed in a vacuum oven at 100 °C for 15 min to crosslink the immobilized DMOAP.

### 2.3. Immobilization of B40t77 Aptamer on DMOAP-Coated Slides

To immobilize B40t77 aptamer on DMOAP-coated slides, we spotted droplets (0.5 µL) of B40t77 aptamer solution (8 µg/mL) on the surface of DMOAP-coated slides in the form of a small array with the help of a micropipette and kept it in a self-made humidified chamber for 1 h. After the immobilization of aptamer, the glass slide was blow-dried under a stream of nitrogen.

### 2.4. Fabrication of B40t77 Aptamer-Based LC Biosensing Platform for gp-120 Detection

Glass slides with immobilized B40t77 aptamer were then incubated with gp-120 solution of different concentrations (8, 4, 2, 1, 0.5, 0.2 and 0.1 µg/mL). After 30 min of incubation in a humidified chamber, the glass slide was rinsed with RNAase-free water and then dried under a nitrogen gas stream. The volume of gp-120 solution used was 0.5 µL.

### 2.5. Selectivity of the Designed B40t77 Aptamer Based LCs Biosensor

The selectivity of the proposed B40t77 aptamer-based LCs biosensor was analyzed in the presence of bovine serum albumin (BSA), human immunoglobulin protein (IgG) and hepatitis A virus capsid protein VP1 (Hep A VP1).

### 2.6. Preparation of LC Optical Cell for Imaging Optical Images

A sample glass slide and DMOAP-coated slide were used to prepare an LC optical cell. The two glass slides were put together and separated by Mylar films (thickness ~6 µm) at both ends of the glass slides. Binder clips were used to secure the LCs optical cell at both ends. Approximately 10 µL of LCs was loaded into the empty space between the two glass slides by using capillary force. After 15 min, the prepared LC optical cell was analyzed using the 1× objective lens under a polarized optical microscope, Nikon ECLIPSE LV100POL (Tokyo, Japan), in the transmission mode.

### 2.7. Atomic Force Microscope (AFM) Characterization

The morphology of different glass slide surfaces was investigated by using the tapping mode with the AFM system (Bruker Dimension Icon atomic force microscope, Singapore, Singapore). In detail, the DMOAP-coated slide, DMOAP-coated slide with immobilized B40t77 aptamer and B40t77 aptamer modified glass slide after addition of gp-120 protein were characterized.

## 3. Results and Discussion

### 3.1. Schematic Illustrations for the Preparation and Sensing Strategy of B40t77 Aptamer-Based LC Biosensor

**Figure 1:**
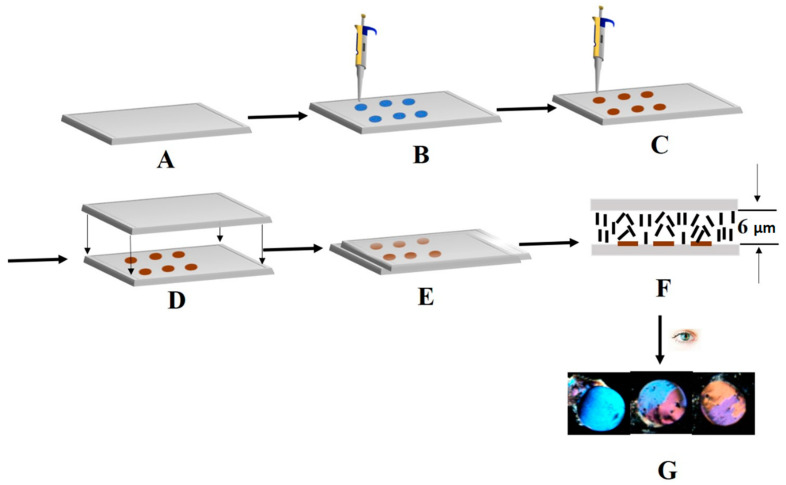
Schematic illustration of the preparation and sensing strategy of proposed bio-sensing approach: (**A**) cleaning and preparation of *N,N*-dimethyl-n-octadecyl-3aminopropyltrimethoxy-silyl chloride (DMOAP)-coated glass slides; (**B**) dispensing droplets of B40t77 aptamer solution in an array form; (**C**) after immobilization of B40t77 aptamer, gp-120 protein droplets were dispensed on immobilized B40t77 aptamer; (**D**) after washing and drying, place spacer (6µm) on left and right edges of slides, followed by covering the slide with another DMOAP–coated glass slide; (**E**) clip the left and right edges of both slides with clippers to make an optical cell; (**F**) introduce liquid crystal (LC) 4-cyano-4-pentylbiphenyl (5CB) into the prepared optical cell; (**G**) visualization under polarized optical microscope with the naked eye, analysis of final results (dark to bright transition in optical appearances).

An appropriate amount of B40t77 aptamer was immobilized onto DMOAP-coated glass slides in the form of a small array with the help of a micropipette (Figure 1). After immobilization, B40t77 aptamer immobilized spot areas were incubated with gp-120 solution. Finally, the fabrication of an LC optical cell was undertaken for the imaging results of the final LC optical responses. While analyzing the final optical images of the LCs, disruption of homeotropic alignment of LCs due to the presence of gp-120 bound to surface-immobilized B40t77 aptamer resulted in the bright images, whereas the dark images were produced due to the LCs homeotropic alignment induced by the orientation agent (DMOAP) coated on the glass slide surface.

### 3.2. Optimization of B40t77 Aptamer Concentration

**Figure 2:**
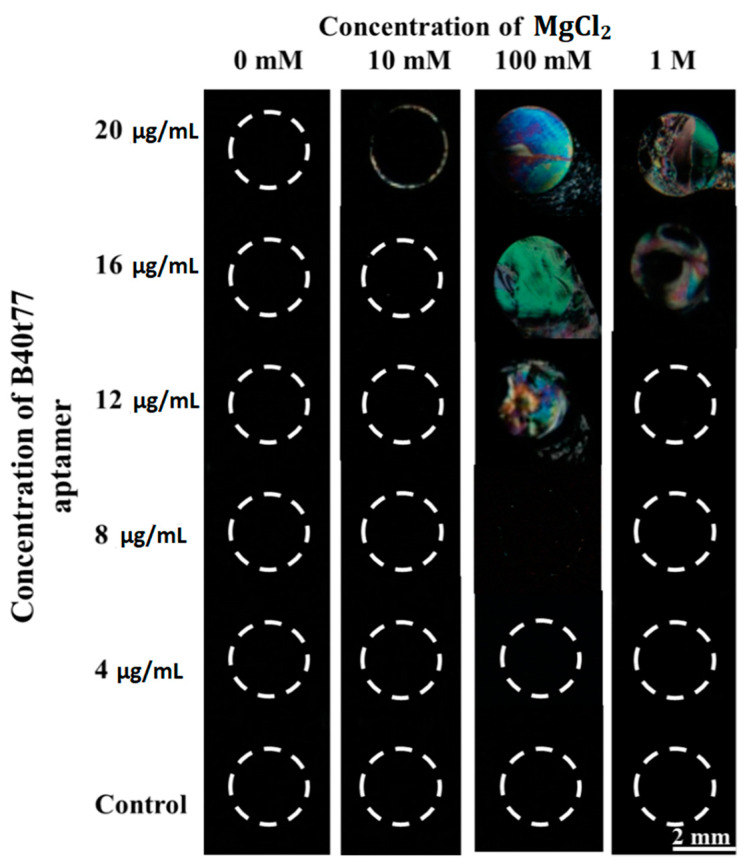
Optical images of LCs supported on DMOAP-coated surface immobilized with B40t77 aptamer solutions made in different MgCl_2_ concentrations; MgCl_2_ concentrations are 1 M, 100 mM, 10 mM and 0 mM; control is just TE buffer with different concentrations of MgCl_2_.

First, we checked the effect of different concentrations of MgCl_2_ on the sensitivity of our recognition element. In this pursuit, we spotted droplets of different concentrations (20, 16, 12, 8 and 4 µg/mL) of B40t77 aptamer solutions made in different concentrations of MgCl_2_ (0 mM, 10 mM, 100 mM and 1 M). As shown in Figure 2, LC optical images with B40t77 aptamer solutions made in 100 mM MgCl_2_ provided the best sensitivity and uniformity. Since the concentration of B40t77 aptamer can also influence homeotropic alignment of LCs, we thereby determined the suitable amount of B40t77 aptamer. In this experiment, to immobilize our recognition element (B40t77 aptamer), we used a DMOAP-coated glass slide as a platform because it has previously been widely used to control the alignment of LCs and for the immobilization of biomolecules [33,34]. To optimize the B40t77 aptamer concentration, we spotted droplets of B40t77 aptamer solution (~0.5 µL) with different concentrations on the DMOAP-coated slide in an array format. This slide was then kept in a humidified chamber for 1 h at room temperature. Finally, after 1 h, the slide was blow-dried to make an LC optical cell.

**Figure 3:**
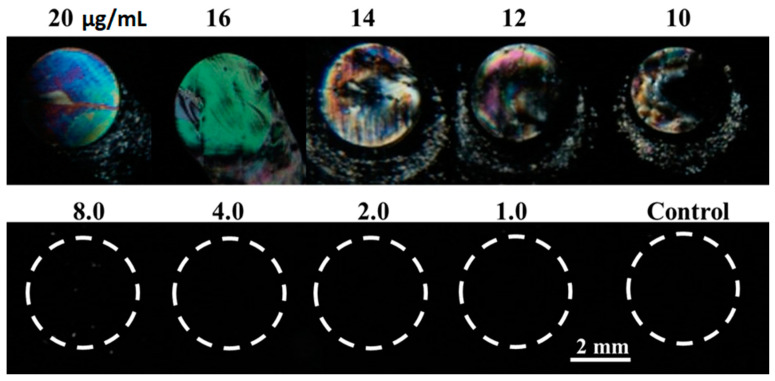
Optical images of LCs supported on a DMOAP-coated slide immobilized with B40t77 aptamer solution with different concentrations; number above each spot is B40t77 aptamer concentration (µg/mL), whereas control is just TE buffer without B40t77 aptamer.

Optical images of LCs under the crossed polarizer are shown in Figure 3. It can be seen that the LCs alignment was disrupted by the surface-immobilized B40t77 aptamer and gave a bright spot when the concentration of B40t77 aptamer was higher than 8 µg/mL. In other regions without any B40t77 aptamer, or in regions where the B40t77 aptamer concentration was 8 µg/mL or lower, the orientation of LC was not disrupted, as is evident by the dark image. These results suggest that 8 µg/mL concentration is the suitable amount of B40t77 aptamer, which ensured the appropriate quantity of the recognition element to immobilize on the glass slides coated with DMOAP for subsequent experiments.

### 3.3. Effect of Time on Immobilization of Recognition Element on DMOAP-Coated Glass Slide Surface

**Figure 4:**
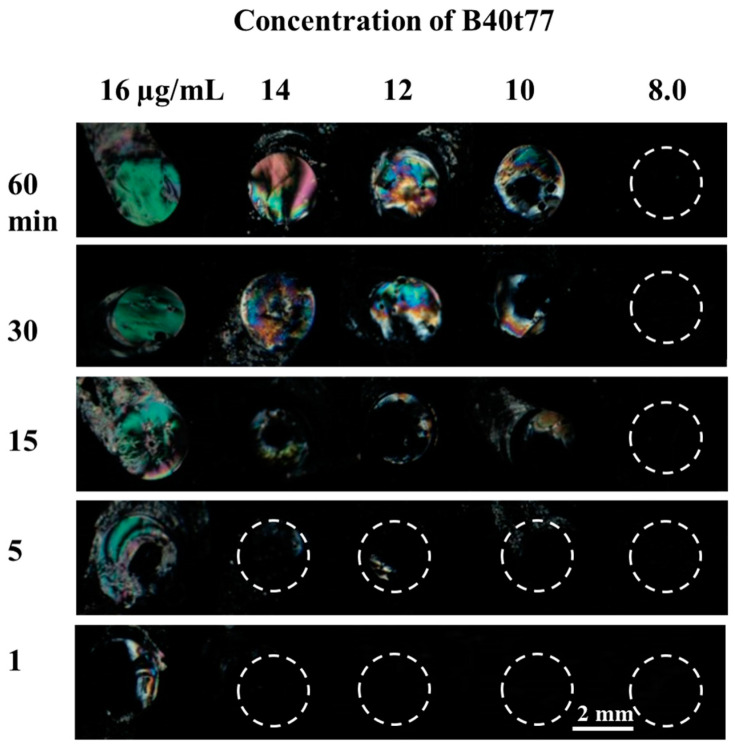
Optical images of LCs supported on a DMOAP-coated slide immobilized with B40t77 aptamer solution of different concentrations showing effects of different immobilization times; the B40t77 aptamer concentrations used are 16, 14, 12, 10 and 8 µg/mL.

To investigate whether time played a role on the immobilization of the recognition element on the surface of the DMOAP-coated glass slide, we dispensed droplets (0.5 µL) of different concentrations (16, 14, 12, 10 and 8 µg/mL) of B40t77 aptamer in a small array and allowed it to incubate for different immobilization times (1, 5, 15, 30 and 60 min). As shown in Figure 4**,** we found the best uniform results when the immobilization time was 60 min. Moreover, we observed that for higher concentration (12–16 µg/mL), incubation time was not the limiting factor, but for proper immobilization in the case of lower concentration, 60 min was the required time for proper immobilization of the recognition element. A shorter incubation time led to improper immobilization of B40t77 aptamer, resulting in weak signals.

### 3.4. Characterization of the Designed Biosensing Platform

**Figure 5:**
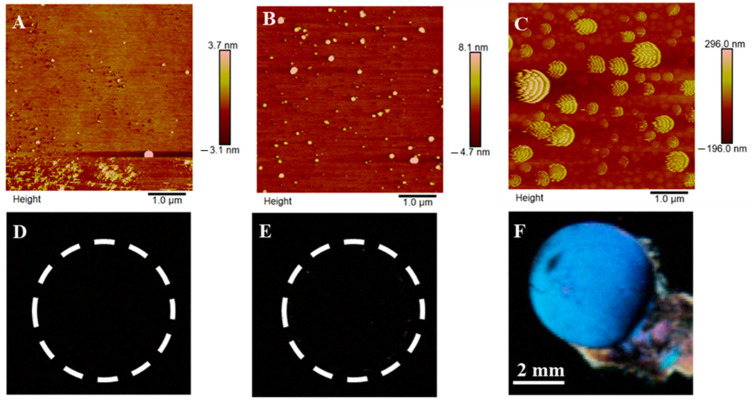
AFM images of different slide surfaces: (**A**) with DMOAP coating; (**B**) after B40t77 aptamer (8 µg/mL) immobilization; (**C**) after addition of gp-120 (8 µg/mL), and the corresponding polarized optical images of LCs supported on different slide surfaces; (**D**) coated with DMOAP; (**E**) with B40t77 aptamer (8 µg/mL) modification; (**F**) after addition of gp-120 (8 µg/mL).

The polarized optical microscope images of the LCs supported on DMOAP-coated slides, after immobilization of appropriate amount of recognition element, with addition of target gp-120 protein and the corresponding AFM images, are shown in Figure 5. In Figure 5D, the dark polarized optical image of LCs shows the homeotropic orientation of LCs induced by the DMOAP coated on the glass slide surface, also confirmed in Figure 5A, showing the AFM image of the corresponding glass slide surface. After successful immobilization of the appropriate amount (8 µg/mL) of B40t77 aptamer on the glass slide, also confirmed by the surface topology changes as shown in the AFM image (Figure 5B), the corresponding optical image of LCs remained almost dark, as shown in Figure 5E. This result clearly showed that such concentration of B40t77 aptamer immobilized on a DMOAP-coated glass slide was not sufficient to disrupt the LC homeotropic alignment. However, after the addition of target gp-120, pronounced changes in the topology were observed (Figure 5C) and resulted in birefringence of the optical image of LCs as shown in Figure 5F. Moreover, we also concluded that this bright response of the LC optical image confirmed the interaction of gp-120 and B40t77 aptamer immobilized on the surface, which was capable of disrupting LCs homeotropic orientation.

### 3.5. Detection of Target gp-120 by Surface-Immobilized Recognition Element B40t77Aptamer

**Figure 6:**
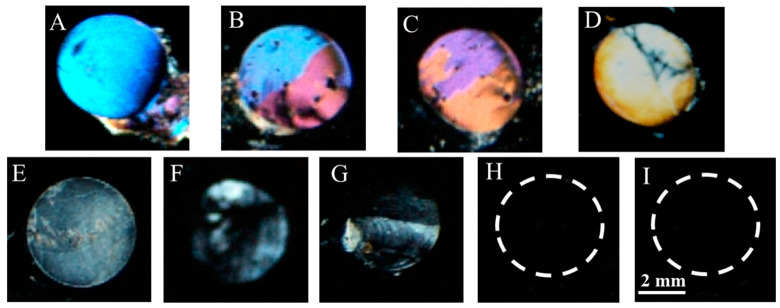
Optical images of LCs supported on B40t77 aptamer (8 µg/mL) immobilized DMOAP-coated glass surfaces after addition of different concentrations of gp-120: (**A**) 8, (**B**) 6, (**C**) 4, (**D**) 2, (**E**) 1, (**F**) 0.5, (**G**) 0.2, (**H**) 0.1 and (**I**) 0 µg/mL, respectively.

After optimization of the appropriate amount of the recognition element, we investigated the viability of the proposed biosensing platform by incubating it with a series of concentrations (8, 4, 2, 1, 0.5, 0.2 and 0.1 µg/mL) of target gp-120 protein. The final polarized optical images of LC optical cells are shown in Figure 6. In previous studies [35,36], the optical appearance of LCs was used as a mean to report target analyte concentration. In the present work, we also observed that a higher concentration (8, 4 and 2 µg/mL) led to a bright signal that can be easily distinguished from a dark LC background, whereas low concentration (1, 0.5 and 0.2 µg/mL) led to a white or gray color signal. However, when the concentration of gp-120 was further reduced to 0.1 and 0 (control) µg/mL, no bright image could be observed, as shown in Figure 6. This result suggests that concentrations below 0.2 µg/mL were not sufficient to disrupt LC homeotropic orientation, resulting in a dark optical image. Therefore, the limit of detection of the proposed biosensing platform was found to be 0.2 µg/mL. Additionally, bright responses of LC optical image confirmed the binding of target gp-120 protein with B40t77 aptamer immobilized on the surface of the proposed LC biosensing platform.

### 3.6. Quantitative Analysis of Biosensor Performance

**Figure 7:**
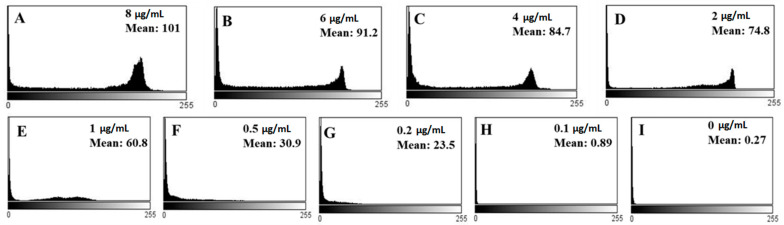
(**A**–**I**) are showing mean gray values corresponding to different optical images of LCs given in Figure 6.

The final results shown in Figure 6 are the optical images of LC optical cells treated with different concentrations of gp-120. These are qualitative results for the detection of targets through the LC biosensor. To quantitatively analyze, we estimated the corresponding mean gray scale intensities of the optical images of LCs using ImageJ software. The different mean grayscale values of the optical images corresponding to different concentrations of gp-120 are shown in Figure 7.

**Figure 8:**
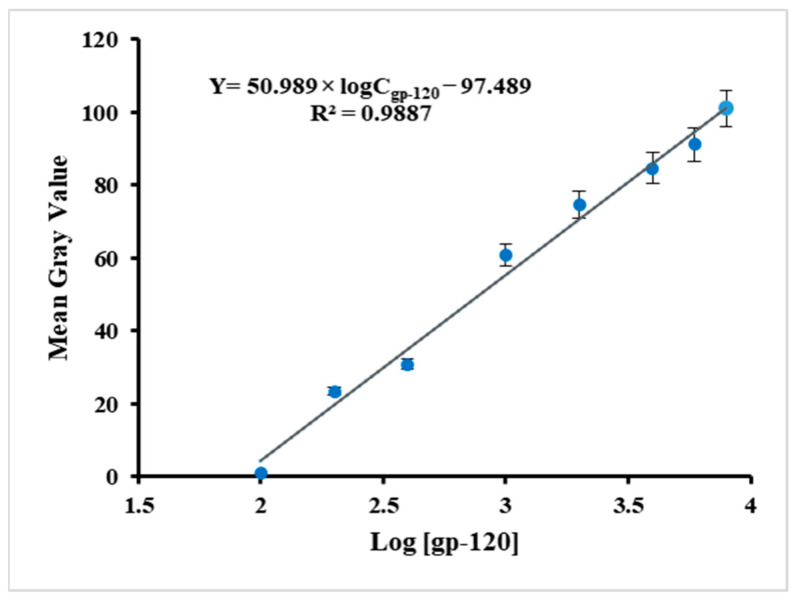
Calibration curve of the mean gray values of LC optical images and the logarithm of the gp-120 concentration (Y = mean gray value of the optical images and C_gp-120_ = concentration of gp-120 (ng/mL)).

These results suggest that the mean grayscale value of the LCs optical image increases with the increase in gp-120 concentration. When the concentration of gp-120 was 8 µg/mL, the mean grayscale value of the LCs optical image was 101 (Figure 7A). With the decrease in gp-120 concentration from 6 to 0.5 µg/mL, the mean grayscale value of the optical images also decreased from 91.2 to 30.9 (Figure 7B–F). The mean grayscale values corresponding to 0.2, 0.1 and 0 µg/mL concentration were 23.5, 0.89 and 0.27, respectively (Figure 7G–I). Additionally, the calibration curve below (Figure 8) shows a linear relationship between the mean gray value of the LCs optical images and the logarithm of gp-120 concentration in the range from 8 to 0.1 µg/mL, where the detection limit of gp-120 in the present biosensing approach was found to be about 0.2 µg/mL. The linear regression equation is

Y = 50.989 × logC_gp-120_ − 97.489 (1)

where Y is the mean grayscale value of the LC optical image, C_gp-120_ is the concentration of gp-120 and the correlation coefficient was 0.9887.

### 3.7. Selectivity

**Figure 9:**
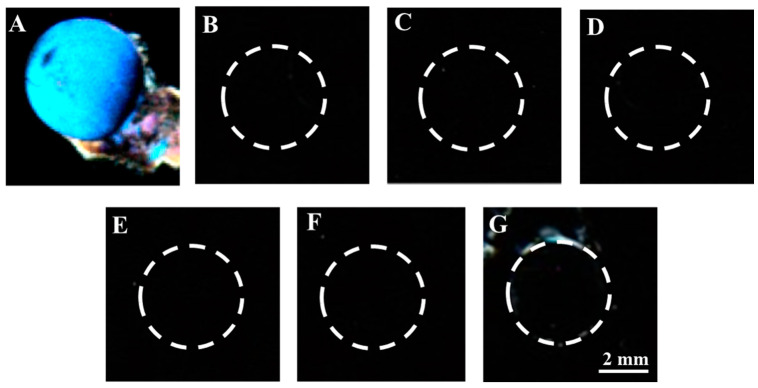
Optical images of LCs supported on B40t77 immobilized DMOAP decorated glass surfaces after treating with different proteins at concentration (8 µg/mL): (**A**) Gp-120, (**B**) BSA, (**C**) Hep A VP1, (**D**) IgG and (**E**–**G**) optical images with double concentration (16 µg/mL) of IgG, Hep A VP1 and BSA, respectively.

To test the specificity of the proposed B40t77 aptamer-based LC bio-sensing platform, we compared the polarized optical microscope images produced by gp-120 and several other targets, such as bovine serum albumin (BSA), human immunoglobulin protein (IgG), and hepatitis A virus capsid protein VP1 (Hep A VP1). All experimental conditions and procedures were the same except gp-120 was replaced by BSA, IgG and Hep A VP1. Concentrations of targets were kept constant at 8 µg/mL. A colorful, bright response was observed in the case of gp-120, as shown in Figure 9A, whereas dark polarized optical images were observed in the case of other targets (Figure 9B–D). To further confirm the selectivity of the biosensor, we also tried the same experimental procedure using a double concentration (16 µg/mL) of other targets and observed no bright response in LCs optical images (Figure 9E–G). However, some weak bright signals were observed at the circumference, which could be due to unbound molecules that were not washed away completely. Therefore, it can be concluded that the proposed biosensing platform demonstrated high selectivity against gp-120.

## 4. Conclusions

In brief, we have developed an aptamer-based LC biosensing platform for the detection of gp-120 by using aptamer B40t77 as a molecular probe and liquid crystal as a signal reporter. The proposed biosensing platform showed high specificity against gp-120. The limit of detection of this bio-sensing platform was found to be 0.2 µg/mL. The present platform was highly specific, simple, low-cost and easy to operate. Moreover, optical image results of this biosensing platform can be quantitatively analyzed through ImageJ software. The developed biosensing approach demonstrates huge promise in overcoming many challenges in medical healthcare, from diagnostics to therapeutics. Furthermore, this biosensing platform has the potential to contribute to the detection of HIV-1 in its early stages.

## Sample Availability

Not available from the authors.
